# Answers to Correspondents

**Published:** 1909-06-05

**Authors:** 


### ANSWERS TO CORRESPONDENTS.

C. F.—The case of diabetes mellitus on which you ask our opinion and advice is decidedly of interest, and we congratulate you upon the progress the patient is making. Under the circumstances it would probably be unwise to alter any part of your treatment, lest the course become less favourable. At the same time, temporary improvements, considerable in degree, are not at all uncommon in diabetes, even when the patients are quite young, and we are afraid that we none the less regard the prognosis as decidedly grave. It is impossible to guarantee that such a patient will live even for months, still less for years. In older persons diabetes often continues for many years, but in young persons, even when there is temporary improvement, the end nearly always comes comparatively soon. More important than the urinary sugar to our minds is the question of acidosis, as tested for by the perchloride of iron test for diacetic acid, or by Legal's test for acetone in the urine, or as actually measured by the amount of combined ammonia in the urine. In a person who is taking aspirin or sodium salicylate there are fallacies in the diacetic-acid test, and we would recommend rather that you employed the sodium nitro-prusside (Legal's) test for acetone periodically. You will find this described in any handbook upon clinical tests, such as French's "Medical Laboratory Methods and Tests" (5s.), or Hutchison and Rainey's "Clinical Methods" (10s. 6d.). So long as your patient has no acetone in her urine, you need not fear danger, even though the sugar should increase. If, as is more likely, she already has acetonuria, we should advise that you send specimens to some laboratory for estimations of the ammonia, say once a fortnight, because the greater the amount of combined ammonia in the 24 hours' urine, the greater the danger of coma.

